# Imaging an Adapted Dentoalveolar Complex

**DOI:** 10.1155/2012/782571

**Published:** 2012-01-19

**Authors:** Ralf-Peter Herber, Justine Fong, Seth A. Lucas, Sunita P. Ho

**Affiliations:** ^1^Division of Orthodontics, Department of Orofacial Sciences, University of California San Francisco, San Francisco, CA 94143, USA; ^2^Division of Biomaterials and Bioengineering, Department of Preventive and Restorative Dental Sciences, University of California San Francisco, San Francisco, CA 94143, USA

## Abstract

Adaptation of a rat dentoalveolar complex was illustrated using various imaging modalities. Micro-X-ray computed tomography for 3D modeling, combined with complementary techniques, including image processing, scanning electron microscopy, fluorochrome labeling, conventional histology (H&E, TRAP), and immunohistochemistry (RANKL, OPN) elucidated the dynamic nature of bone, the periodontal ligament-space, and cementum in the rat periodontium. Tomography and electron microscopy illustrated structural adaptation of calcified tissues at a higher resolution. Ongoing biomineralization was analyzed using fluorochrome labeling, and by evaluating attenuation profiles using virtual sections from 3D tomographies. Osteoclastic distribution as a function of anatomical location was illustrated by combining histology, immunohistochemistry, and tomography. While tomography and SEM provided past resorption-related events, future adaptive changes were deduced by identifying matrix biomolecules using immunohistochemistry. Thus, a dynamic picture of the dentoalveolar complex in rats was illustrated.

## 1. Introduction

The load-bearing bone is a dynamic tissue and continuously adapts to changes in loads [1]. In the periodontium, the cementum of a tooth is attached to the alveolar bone by the periodontal ligament (PDL), and the root is contained within the alveolar bone socket. Cementum and bone are calcified tissues of similar chemical composition, but cementum is far less dynamic [2]. The vascularized and innervated PDL consists of basic constituents that resist and dampen mechanical loads. Different types of collagen and noncollagenous proteins including polyanionic water attracting molecules, the proteoglycans (PGs), all of which accommodate cyclic occlusal loads of varying magnitudes and directions. Unlike other ligaments within the musculoskeletal system, the blood vessels in the PDL are continuous with blood vessels in the endosteal spaces of bone [3]. Although PDL and bone are two dissimilar tissues in physical and chemical properties, the continuity formed by blood vessels enables a flow of nutrients and maintains cellular activity responsible for PDL turnover and bone remodeling and modeling. Development and growth superimposed with functional loads [4] may cause posterior lengthening of the rat jaw [5], and can contribute to PDL turnover, bone remodeling, and load-related modeling during the lifespan of a rat. As a result, rat molars are thought to exhibit an inherent distal drift [6], but this theory continues to be controversial [7, 8]. Regardless, the drift of the molars causes bone resorption located on the distal side of the root and bone formation on the mesial side. In this study, the distal side of the root and the adjacent alveolar bone will be referred to as the distal root bone complex (bone resorption side), and the mesial side of the root and adjacent bone as the mesial root bone complex (bone apposition side). Specific to this study are the various imaging modalities implemented to investigate the physical, chemical, and biochemical changes reflective of distal drift in a rat bone-PDL-cementum complex.

Numerous studies in dental research have used the rat periodontium as a model to investigate adaptation of bone, PDL, and root due to perturbations, such as disease [9] and extraneous loads [10]. The perturbations could affect the bone-PDL and cementum-PDL attachment sites. Hence, it is important to know the baseline parameters in the rat model before additional variables are imposed. In this study, we present an overview of commonly used imaging methods to investigate calcified tissues and the PDL, while addressing the plausible artifacts during specimen preparation, imaging, and postprocessing of experimental data. 

Micro X-ray imaging is a popular method, as it provides a three-dimensional (3D) representation of organs and tissues. Micro X-ray imaging is used to study the internal architecture of bone [11], tooth [12], and the bone-PDL-cementum complex [3], along with resorption-related changes of the root [13]. Additionally, X-ray attenuation maps can be related to mineral density variations within calcified tissues [11]. 

Scanning electron microscopy (SEM) is used to study tissue architecture at a relatively higher resolution. In this study, SEM was used to identify resorbed bone [14] and root [15] morphology. Although not used in this study, the higher resolving power of a transmission electron microscope (TEM) provides information about the inorganic crystal type and morphology within a tissue matrix [16]. While most conventional SEM and TEM operate under high vacuum mode, an atomic force microscope (AFM) can image site-specific regions within tissues at ambient conditions, facilitating nanoscale and microscale observations of tissue architecture under hydrated conditions [17] with minimum specimen preparation [18]. AFM coupled with a nanoindentation transducer can be used for mapping site-specific mechanical properties of tissues and their interfaces [3]. 

Various spectroscopy techniques, including Fourier transform infrared spectroscopy (FTIR) [19] and Raman microspectroscopy [20] provide chemical composition of calcified tissues. Complementing spectroscopy techniques are numerous conventional histological, and immunohistochemical stains to identify cells relative to the spatial localization of biomolecules of interest. Histological analyses specific to this study include, hematoxylin and eosin (H&E) [21, 22], tartrate-resistant acid phosphatase (TRAP) [23, 24], and immunohistochemical staining using fluorescent probes for receptor activator of nuclear factor *κ*B ligand (RANKL) [25, 26] and osteopontin (OPN) [27, 28]. Fluorochrome labeling is another widely used technique to study the temporal growth of bone and cementum [29, 30]. 

 In this study, micro X-ray computed tomography (Micro-XCT) was used to image and measure the anatomical, physical, and chemical properties of calcified tissues in 3D and locate resorption and remodeling related events. Micro-XCT data was complemented with (immuno)histochemical studies to investigate biomolecular events within the bone-PDL-cementum complex. Furthermore, a combination of these techniques performed consecutively on the same specimen allowed correlating 2D histological sections with 3D tomography, performed before preparing the specimen for histology. Thus, synergetic effects of imaging modalities were exploited to develop a dynamic picture of the resorption and remodeling-related events in the load-bearing bone-PDL-cementum complex. The combination of techniques within describes complex events in the periodontium and illustrates potential mechanisms elucidating cause-and-effect relationships. 

## 2. Materials and Methods

Maxillae from 7-week to 4-month-old male Sprague Dawley rats were used. Rats were obtained using animal tissue transfer according to guidelines of Institutional Animal Care and Use Committee (IACUC), University of California San Francisco (UCSF). 

### 2.1. Micro X-Ray Computed Tomography

Entire heads or hemimaxillae were imaged using Micro-XCT (Micro XCT-200, Xradia, Pleasanton, CA). The occlusion was imaged using whole heads, while the tooth-bone complex was imaged using hemimaxillae. After harvesting, all specimens (*N* = 8) were placed in polymeric containers with 70% ethanol, mounted on a specimen stage, and imaged at different magnifications and power as needed using a Micro-XCT. Polymeric wire was used to bring the upper and lower jaws together to approximate the occlusal plane and was imaged using a 2x objective, at 90 KVp and a power of 6 W. The maxillae per se were imaged at 2x and 4x and 75 KVp and a power of 6 W. Each tomography was reconstructed from 3500 radiographic projections obtained from a full circle of 360° and exposure times were adjusted to yield 6000 to 8000 counts per pixel of each recorded radiograph approximating 25% of the original X-ray intensity passing through the specimen and arriving at the detector. Associated tomographies were reconstructed using reconstruction software (XMReconstructor, Version 7.0.2817, Xradia Inc., Pleasanton, CA). 3D images were postprocessed using the Xradia 3D viewer and Amira software (Visage Imaging Inc., Version 5.2.2, San Diego, CA).

### 2.2. Scanning Electron Microscopy

Maxillary molars (*N* = 3) were isolated using forceps, and the remaining bony sockets were cut to separate bone from the mesial and distal complex. The exposed alveolar socket and molars were mounted on SEM stubs and sputtered with gold. The specimens were examined using an SEM (S4300, Hitachi, Tokyo, Japan) with an electron energy of 5 keV. 

### 2.3. Histology

Intact hemimaxillae (*N* = 5) were decalcified in 0.5 M EDTA solution for 3 weeks. The specimens were dehydrated with 80%, 95%, and 100% Flex alcohol (Richard- Allan Scientific, Kalamazoo, MI) before embedding in paraffin (Tissue Prep-II, Fisher Scientific, Fair Lawn, NJ). They were sagittally sectioned on a rotary microtome (Reichert- Jung Biocut, Vienna, Austria) using a disposable steel blade (TBF Inc., Shur/Sharp, Fisher Scientific, Fair Lawn, NJ). The paraffin serial sections were mounted on Superfrost Plus microscope slides (Fisher Scientific, Fair Lawn, NJ) and deparaffinized with xylene.

#### 2.3.1. Hematoxylin and Eosin Stain

The sections were stained with hematoxylin (Fisher Scientific, Kalamazoo, MI) and eosin (Fisher Scientific, Kalamazoo, MI) [31]. The stained tissues were characterized using a light microscope (BX 51, Olympus America Inc., San Diego, CA) and analyzed using Image Pro Plus v6.0 software (Media Cybernetics Inc., Silver Spring, MD). 

#### 2.3.2. Tartrate-Resistant Acid Phosphatase Histochemistry

Deparaffinized serial sections were used for TRAP staining. In brief, the method [32] included treating the rehydrated specimens with 0.2 M acetate buffer, a solution of 0.2 M sodium acetate and 50 mM L(+) tartaric acid (Sigma-Aldrich, St. Louis, MO). After 20-minute incubation at room temperature, naphthol AS-MX phosphate and fast red TR salt were added followed by incubation at 37°C for 1 hour. The stained sections were washed in deionized water, counterstained with hematoxylin, and mounted with Immu-Mount (Thermo Scientific, Fremont, CA) for subsequent examination under a light microscope as stated above. Multiple images were stitched together to produce the resulting figure using Microsoft Research Image Composite Editor (Microsoft Corporation, Redmond, WA).

#### 2.3.3. Immunostaining

In the method used [26], deparaffinized sections were rehydrated, digested with trypsin (Sigma-Aldrich, St. Louis, MO) for ten minutes at 37°C, and subsequently rinsed and washed in deionized water. Specimens were incubated in blocking buffer (3% goat serum, 0.1% BSA in 1x PBS) and then in primary antibodies polyclonal rabbit anti-RANKL (Santa Cruz Biotechnology Inc., sc-9073, Santa Cruz, CA) or monoclonal mouse anti-OPN (Santa Cruz Biotechnology, Inc. Akm2A1, Santa Cruz, CA). Primary antibodies were diluted to 1 : 50 in blocking buffer. Slides were stored at 4°C in a humid case overnight, followed by washing three times for five minutes with 0.1% Tween-20 in PBS (PBST) and then incubated with secondary antibodies. Alexa Fluor 594 goat anti-rabbit (Invitrogen, A-11029, Carlsbad, CA) was used to label polyclonal rabbit anti-RANKL and Alexa Fluor 488 goat anti-mouse (Invitrogen, A-11037, Carlsbad, CA) to label monoclonal mouse anti-OPN, at 1 : 300 (diluted in blocking buffer). Slides were incubated in a opaque humid case for one hour at room temperature. Sections were washed three times for five minutes with PBST and then stained with 1 : 10000 trihydrochloride trihydrate (Invitrogen, Carlsbad, CA) for ten minutes in the absence of light. Slides were rinsed twice with PBS and mounted using Fluoro Gel (Electron Microscopy Sciences, Hartfield, PA). Stained tissues were visualized using Eclipse E800 fluorescent microscope (Nikon Inc., Melville, NY). TRITC filter (540–565 nm) was used to excite Alexa Fluor 594 (abs. 590 nm, emit. 617 nm), FITC filter (465–495 nm) to excite Alexa Fluor488 (abs. 495 nm, emit. 519 nm), and DAPI filter (340–380 nm) to excite trihydrochloride trihydrate (abs. 358 nm, emit. 461 nm). Multiple images were stitched together as described above.

### 2.4. Fluorochrome Study

Under regulation of the animal protocol No. AN083692 and AN080608-02 approved by the IACUC, UCSF, 6-week- (*N* = 3) and 4-month- (*N* = 3) old male Sprague-Dawley rats were given intraperitoneal injections with alternating tetracycline hydrochloride and alizarin red (both Sigma-Aldrich, St. Louis, MO) on days 0, 3, and 7. According to the method used [29], 25 mg fluorochrome per 1 kg rat body mass was diluted in 2% NaHCO_3_ to a concentration of 0.01 mg/*μ*L before intraperitoneal injection. On day 8, rats were sacrificed using CO_2_ gas and bilateral thoracotomy. Maxillae were dissected, fixed in 4% paraformaldehyde overnight, sectioned sagittally using a low-speed diamond saw (Isomet, Buehler, Lake Bluff, IL), and ground into 50 *μ*m thick specimens for viewing under the fluorescent microscope (Eclipse E800, Nikon Inc., Melville, NY). FITC filter (465–495 nm) was used to excite tetracycline HCl (abs. 390–425 nm, emit. 525–560 nm) and TRITC filter (540–565 nm) to excite Alizarin Red (abs. 530–560 nm, emit. 580 nm). Multiple images were stitched together as described above.

## 3. Results and Discussion

In the 19th century, Wolff discussed adaptation of bone due to mechanical forces [1]. The occlusal force, primarily used in grinding the hard diet fed to rats, is the most prominent force in the periodontium [33]. Within this adaptation lies growth and function-related changes in bone and cementum, which will be illustrated through various imaging modalities. 

**Figure 1:**
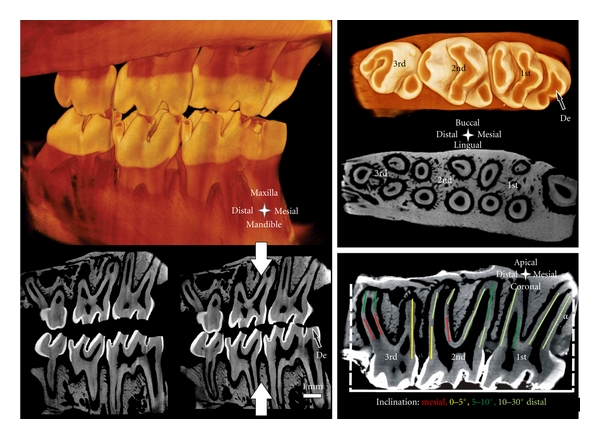
Left top: Simulated occlusion of a left rat maxilla with the corresponding mandible imaged with Micro-XCT. Left bottom: sagittal sections simulate open (left) and closed (right) bite. Top right: occlusal surface and transversal section of a right maxilla. Bottom right: sagittal section; note the predominant distal inclination of the roots. Inclination *α* is measured for the most mesial root as demonstrated. The colored segments on the roots represent the inclination angle of the roots. De = exposed dentin.

Micro-XCT is a noninvasive technique that requires minimum specimen preparation. Specimens can be imaged under wet conditions, preserving different tissue structures and at microscopic resolution below 5 microns. Tomographies and virtual scans can be processed to evaluate mineral density, resorption volumes, displacement fields, and 3D spatial association of the root with the bony socket. The 3D tooth-bone association can provide insights to form-function relationships. Additionally, 2D images recorded with other techniques including light microscopy can be related to the 3D tomographies and 2D virtual sections, and potential artifacts due to specimen preparation can be identified. In this study, we used Micro-XCT for *in situ* imaging, to approximate interdigitation of disarticulated maxilla and mandible of a rat by imaging at 2x magnification (Figure 1, left). Root morphology, and resorbed pits in bone and roots were measured. Structural analysis through the volume of a specimen was correlated with virtual serial sections with least interpolation. Additionally, X-ray attenuation indicative of mineral density variations at the bone-PDL interface was investigated.

**Figure 2:**
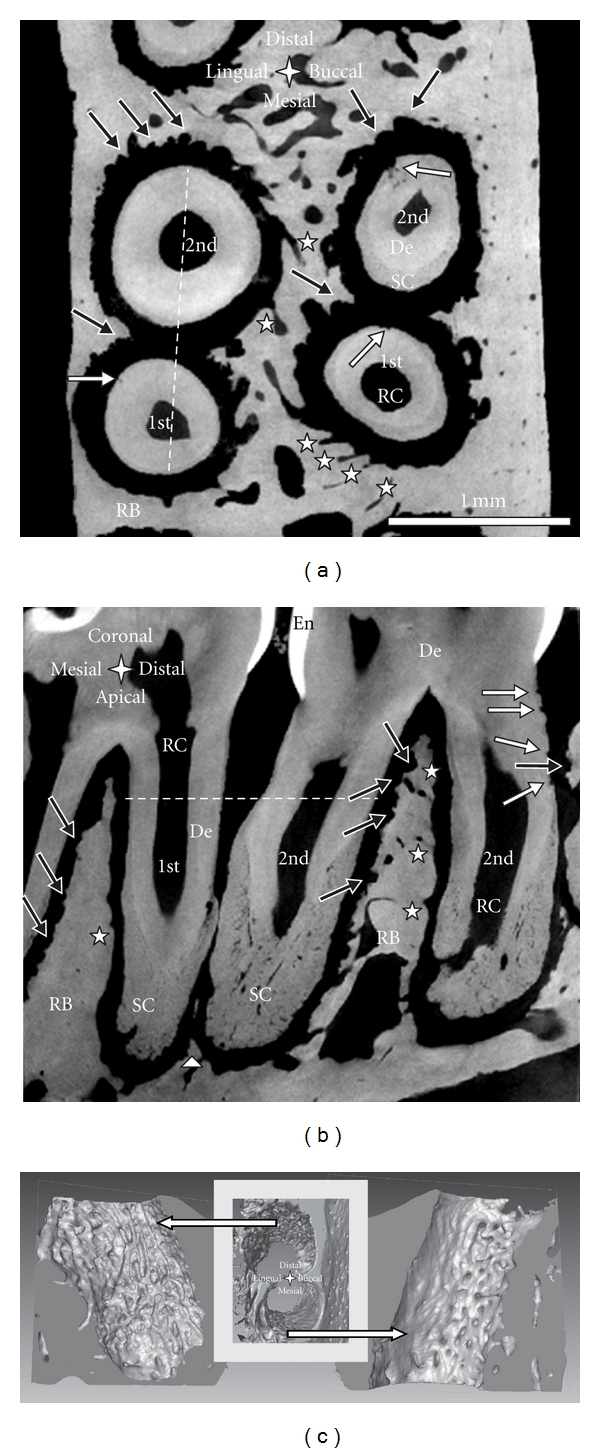
Transverse (a) and sagittal section (b) of the distal root of a 1st and the mesial root of a 2nd molar of a right maxilla exhibiting minimal interdental bone imaged with Micro-XCT; the white dotted lines indicate the position of the other section, respectively; note: bone resorption (black arrows), root resorption (white arrows), blood vessels (white stars), and residual interdental bone (white triangle). (c) 3D images of alveolar bone of the buccal roots in (a); bone surface of the distal root-bone complex shows resorption tracks (left image); holes on bone surface of the mesial complex indicate entering of blood vessels (right image). De = dentin, RB = interradicular bone, SC = secondary cementum, RC = root canal, and En = enamel.

**Figure 3:**
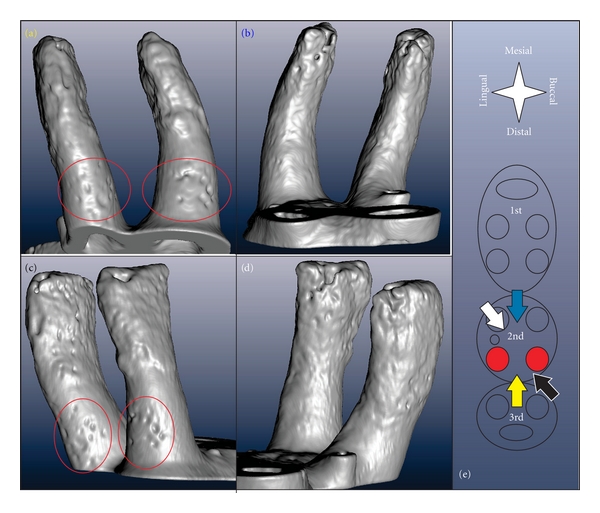
3D reconstruction of the distal roots of 2nd molar viewed from different directions indicated by corresponding colors in (e). Accordingly, (a) shows the distal (yellow arrow), (b) the mesial (blue), (c) the buccal-distal (black), and (d) the lingual-mesial (white) surface of the roots; the apical parts of the roots are rough while the coronal parts are regularly rounded except for the resorption pits located on the coronal distal side of the root indicated by the red ovals.

 The Micro-XCT images presented in Figures 1–3 illustrate an accurate anatomy of the dentoalveolar complex. Accuracy is necessary to spatially correlate 2D measurements from other complementary studies by identifying landmarks, and anatomical planes within the dentoalveolar complex. Furthermore, 2D and 3D data will help identify deviations due to external perturbations such as, disease or load-mediated influences from baseline measurements. 

A rat hemimaxilla contains one incisor and three molars. We focused our investigation on the molars responsible for masticatory function. The crown of the 1st molar is the biggest, with the largest occlusal surface, the 2nd molar measures approximately 2/3 s, and the 3rd molar 3/5 s of the length of the 1st molar. In all specimens, a significant amount of occlusal wear was commonly observed. Figure 1 demonstrates enamel wear and exposed dentin on the occlusal surface. This could lead to varying contact between opposing teeth and is a potential cause for altered biomechanics and modeling-related adaptation in the bone-PDL-cementum complex throughout the lifespan of an organism.

The 1st molar is located mesially and the 3rd molar distally. Often times the challenge lies in identifying the anatomical directions of the specimen when only a part of it is imaged. Hence, certain predefined anatomical features are used to assign anatomical directions, and planes. Lingual and buccal sides of the hemimaxilla denote the tongue and cheek-sides, respectively. The bone around the molars is slightly curved with the center of the curve toward the lingual side. The occlusal surface of all molars contains 2 or 3 enclosed depressions, with the most mesially located in the lingual half. The roots on the lingual side are more uniform in appearance and more closely aligned. The 1st, 2nd, and 3rd molar have 5, 4, and 3 roots, respectively. The 3rd molar exhibits an additional, but smaller, 4th root. The majority of the roots are within 2 parallel planes, the lingual and buccal planes from the midsagittal section. The roots exhibit a slight distal inclination *α* of 10°–15°. The angle of inclination is measured at the root apex, as the angle between root surface, and the normal to the occlusal surface, as shown in Figure 1 bottom right. According to the theory of Wolpoff [34], inclinations promote distal drift of the molars. At the age of 4 months, the roots are not straight but exhibit mesial curvature along with increasing distal inclination towards the root apex (Figures 1–3). The mesial root of the 1st and the distal root of the 3rd molar are exceptional cases, as they are centrally located with significant distal and mesial inclinations, respectively. Given such an anatomy, the rate of distal drift can also change due to a change in occlusal forces. Occlusal forces can generate distal force vectors because of the distal inclination of the roots [34]. Interestingly, root inclination in humans and primates is mesial [8, 35] and a physiological mesial drift is reported that enables closing of gaps due to development and function [34]. 

Based on the morphology of the tooth-bone complex in rats, it is conceivable that occlusal loads will compress the PDL in the distal root-tooth complex and simultaneously result in PDL tension within the mesial complex. Similar effects exist in orthodontics, where compression sites in the PDL promote resorption and tension sites promote formation, resulting in tooth migration along the dominant force vector [36]. Cyclic compression and tension of the PDL during mastication could promote bone resorption and formation, respectively. Furthermore, it has been shown that different mechanical demands on the tooth-bone complex [4], and compression and tension sites related to distal inclination of the roots (Figure 2), affect microscale structure of alveolar bone and macroscale form of the bony socket. Consequently, bone morphologies in the distal and mesial root-tooth complex of the same root are inherently different as demonstrated in Figure 2. 

**Figure 4:**
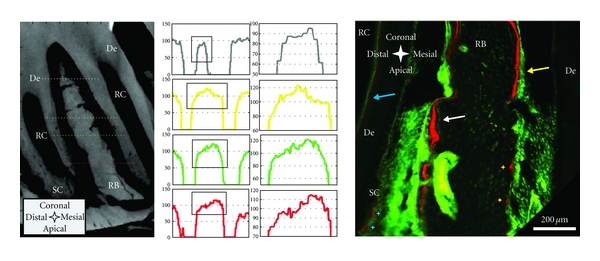
Left panel: Attenuation profiles (arbitrary units) from left to right through interradicular bone; bone formation in the mesial root-bone complex (left side) to bone resorption in the distal complex (right side) of the same molar. Attenuation of the “old” bone on the resorption side (right side) is generally higher and transition from PDL is steeper compared to the newly formed bone on the other side (left side). Right panel: sagittal section of a 7-week-old specimen with fluorochrome labeling; bone deposition predominantly in the mesial root-bone complex (white arrow), but also probably growth-related deposition in the distal complex (yellow arrow); note: predentin formation in the pulp (blue arrow), red stain on resorption pits (yellow stars); formation/repair of secondary cementum (blue stars). De = dentin, RB = interradicular bone, RC = root canal, and SC = secondary cementum.

**Figure 5:**
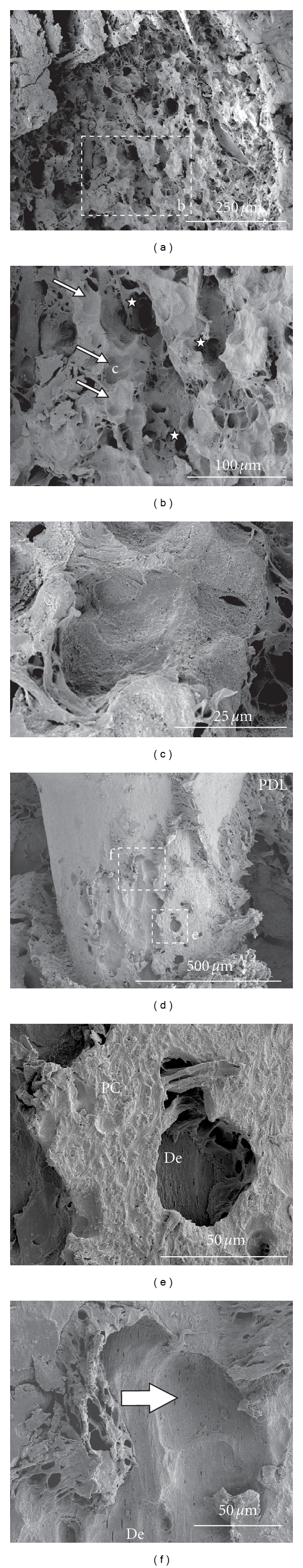
(a–c) SEM image of bone from the distal root-bone complex; (b) note: resorption pits (white arrows), blood vessel space (white stars); (c) high magnification of resorption pit. (d–f) SEM image of a root exhibiting heavy root resorption; (e) isolated pit in primary cementum; (f) larger resorption pit subdivided into smaller ones; note tubular structure of dentin in pits of (e) and (f). De = dentin, PC = primary cementum, and PDL = periodontal ligament.

**Figure 6:**
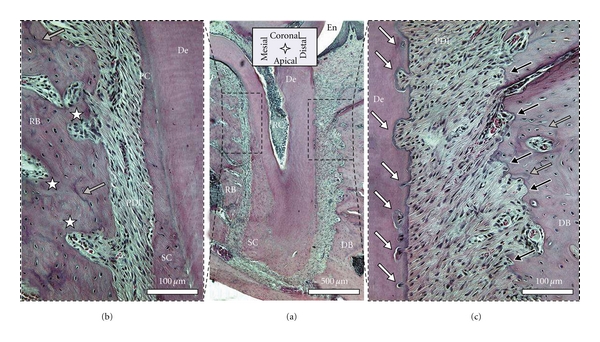
Histological sections stained with H&E: (a) shows the entire root and indicates the position of the mesial (b) and distal (c) root-bone complex at higher magnifications; rough pitted bone surface on the surface of the distal complex and the regular surface in the mesial complex; note: resorption pits in bone (black arrows), cement lines in bone (grey arrows), root resorption (white arrows), and blood vessel spaces (white stars). De = dentin, RB = interradicular bone, DB = interdental bone, SC = secondary cementum, and PC = primary cementum.

**Figure 7:**
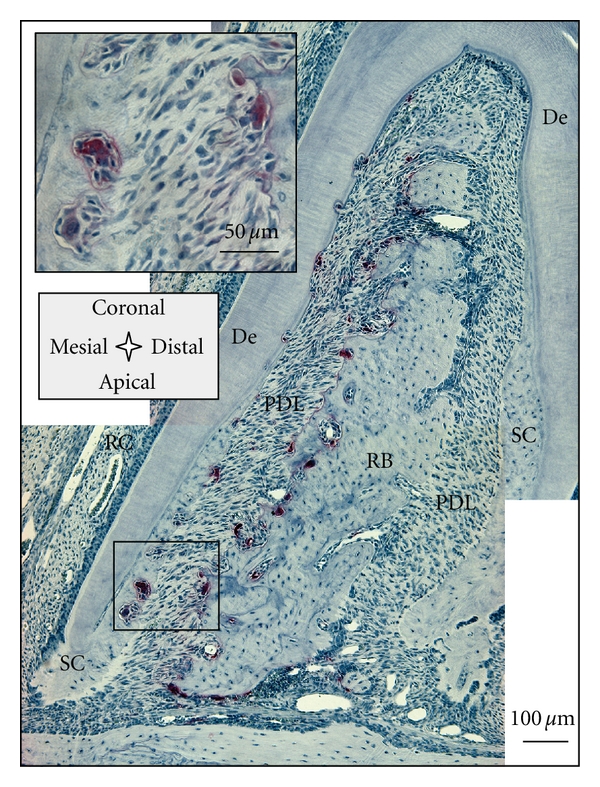
TRAP-positive cells located exclusively in the distal root-bone complex; *insert* shows multinucleated cells resorbing bone and secondary cementum, osteoclasts, and odontoclasts, respectively. De = dentin, RB = interradicular bone, PDL = periodontal ligament, SC = secondary cementum, and RC = root canal.

Bone of the distal complex contains concave-rounded pits separated by narrow sharp ridges, resulting in a rough and pitted surface. This appearance is characteristic for distal bone and originates from the osteoclastic resorption activity also demonstrated in SEM micrographs (Figure 5) and histological images (i.e., Figures 6 and 7) of individual pits approximately 50 to 100 *μ*m in diameter [37]. A reconstruction of the bony socket from the same Micro-XCT scan is illustrated in Figure 2(c). The extent of the osteoclastic activity can be observed as several resorption channels cut through the volume of the bone (Figure 2(c) left). Contrastingly, the bone from the mesial root-bone complex exhibits a smooth surface with convex-rounded bony protrusions into the PDL-space. The bony protrusions are separated by recesses or channels (Figure 2). The channels can be related to blood vessel spaces with red blood cells as shown in the H&E stained sections (Figure 6). The convex protrusions can be attributed to bone formation as demonstrated in the fluorochrome study (Figure 4 right). Regardless, the 3D images of bone demonstrates continuity of blood vessels in the PDL to those in bone (Figure 2(c)). Several histology sections in this study also support this observation in particular, Figure 6, top right.

 The roots exhibit a significant structural anisotropy. The root surface, separated by a 100 *μ*m thick PDL from the resorbed bone in the distal root-bone complex also exhibits resorption pits (Figure 2). The 3D images of the distal roots of a second molar in Figure 3, viewed from different angles illustrates the distribution of resorption pits on the root. Resorption pits on the root are less frequent than on bone. Pits on the root are small, and can be identified predominantly in the coronal region in primary cementum. While the mesial surfaces of the coronal thirds/halves of the roots typically show a regular morphology, several pits were identified in the coronal-distal portion of the roots. The apically located secondary cementum shows an overall high roughness and minor pits on all sides. The rough appearance of the secondary cementum in the apical part of the root has already been reported for rats [38], and was attributed to increased resorption and formation-related activities stimulated by occlusal loading.

Another remarkable anatomical feature is thinning of interdental bone, as shown in Figure 2. The distal root of the 1st molar and the mesial root of the 2nd molar share the same PDL-space and are in physical contact. Interdental bone commonly reaches the cervix of the tooth, and is comparable to the interradicular bone between the distal and mesial roots of the 2nd molar. Previous studies reported partial thinning of interdental bone, and resulting root proximity [39]. Figures 1 and 2 illustrate the apical parts of 2nd and the 3rd molars not separated by bone (Figure 1 bottom right, Figure 2(b)). In general, adequate interdental bone and PDL-space are maintained through combined resorption (distal) and apposition (mesial) related events, accomodating movement of the molars [8]. Based on our observations and previous reports by others, we suggest different migration rates of the individual molars [40].

Apposition and resorption-related events manifest into lower and higher X-ray attenuation profiles in the mesial and distal root-bone complex. Micro-XCT techniques measure X-ray attenuation differences that can be directly related to bone mineral density when calibrated with phantoms of known mineral density [11]. Furthermore, mineral density differences can be exploited from virtual scans at a spatial resolution equivalent to the magnification at which the specimen was scanned. In this study highly attenuating regions in the specimen appear brighter and are related to higher mineral content. In the sagittal and transversal sections of the Micro-XCT images, we consistently observed darker areas representing lower X-ray attenuation close to the PDL interface in bone of the mesial root-bone complex (Figure 4 left). The graphs demonstrate that attenuation of bone in the distal complex is generally higher and that the increase of attenuation from PDL to bone is steeper than in the mesial root-bone complex. Lower attenuation in bone is caused by lower degree of mineralization and/or crystallinity and can be related to the earlier stages of modeled bone associated with distal drift. This continuous apposition of bone in the mesial root-bone complex accompanied by resorption of bone in the distal complex, coupled with adaptations in primary and secondary cementum, is necessary to maintain a uniform functional PDL-space and accommodate the hard pellet diet in rats. 

Complementing X-ray attenuation profiles, are results from fluorochrome labeling. Despite the cumbersome nature of the fluorochrome labeling technique, which includes injecting the animal periodically with different fluorescent dyes, followed by harvesting, specimen preparation, and imaging, the technique illustrates the dynamic nature of bone indicating potentially loaded areas in both tension and compression [41]. Fluorochrome labeling is an effective method to study biomineralization-related events [42]. Fluorochrome dyes form chelate complexes with exposed apatite within a mineralizing tissue. As a result, the fluorescent label demarcates active mineralizing fronts exhibiting mineralized tissue formation at the time of administration. By using alternating dyes and injecting at different timepoints, the stratified growth of bone can be temporally mapped [30]. Figure 4 shows a section of interradicular bone of a 2nd molar from a 7-week-old specimen. The sequence of green-red-green lines in the mesial root-bone complex demonstrates bone formation. The space between two lines shows that the mineralization front moved approximately 10–30 *μ*m in the 3-4 days between two injections, which corresponds to a distal drift of approximately 20–30 *μ*m over the same period [43, 44]. Coronal bone in the distal complex also shows fluorochrome labeling. Furthermore, the surface appears to be regular and convex. This could indicate that bone apposition/repair occasionally also occurs in the distal root-bone complex. However, in the older 4-month-old specimen, significant bone formation was observed in the mesial complex.

 Changes from resorption to apposition activity are the origin of cement lines [41], shown as basophilic lines in H&E stained sections (Figure 6). However, remaining distal bone illustrated a pitted surface, and no fluorochrome labeling, indicating resorption activity. Some of the pits exhibited a red lining on the surface, probably indicating local repair. Furthermore, the distal root in Figure 4, shows regular deposition of predentin in the pulp chamber and minor repair/formation activity on secondary cementum.

Specimen preparation for SEM is more cumbersome than for Micro-XCT. In particular, dehydration, fracturing, and sputtering of the specimen along with imaging under high vacuum can induce several artifacts and affect structural integrity. High-energy electrons can result in disintegration of soft tissue, and the vacuum chamber limits *in situ* experiments. However, its spatial resolution and magnification range are superior to the other techniques presented in this study. Hence, SEM measurements were conducted to study bone and root morphology, and in particular, resorption morphology at higher resolution.  Figure 5(a) shows an SEM image of bone from a distal root-bone complex. At higher magnification, resorption pits and blood vessel openings can be identified (Figures 5(b)-5(c)). In Figure 5(c), a single pit of less than 50 *μ*m was observed. Figures 5(d)–5(f) illustrate a case of excessive root resorption on the distal side of a 2nd molar, with a pattern of large and small pits and regular cementum surface. At a higher magnification, larger pits were subdivided into smaller pits with diameters of approximately 50 *μ*m, (Figure 5(f)). Inside the larger pits within primary cementum (Figure 5(e)), typical tubular structure of dentin was observed. 

While Micro-XCT, SEM, and fluorochrome staining coupled with fluorescence microscopy identified adaptation of the calcified tissue, the distribution of the cells, proteins, and organic matrix could not be imaged sufficiently due to low-contrast for X-rays and high-energy electrons, respectively. Though highly attenuating stains like phosphotungstic acid, osmium tetraoxide, and gallocyanin-chromalum improve imaging of the PDL fibers and most likely cells [45], the information that can be gathered from imaging following staining is still very limited compared to conventional histology and immunohistochemistry. Furthermore, the resolving power of the Micro-XCT is another limit. The latter would not pose a problem for SEM, but this method will introduce artifacts in the organic tissue due to specimen preparation, high vacuum, and higher-energy electrons as stated earlier. Hence chemically fixed histological sections were stained conventionally or using immunolabeling techniques. The preparation of the histological specimens is very time consuming, as it requires fixation, chemical processing, embedding, and sectioning. Moreover, it can introduce artifacts like delaminated interfaces and loss of structural integrity. The identification of stained organic matter is limited by the resolution of the analytical instruments: the optical or fluorescent microscopes. Furthermore, it should be noted that the sections prepared are 2D and can lead to misinterpretation of 3D structures, despite the interpolation between serial sections. 

Though H&E is a conventional stain, it is of value to the study, as it allows to distinguish basophilic structures that stain blue (nuclei), and eosinophilic structures that stain pink (intra and extracellular proteins), or red (red blood cells). This stain gives good structural contrast, and therefore the H&E stained sagittal section in Figure 6 can be compared to sagittal sections imaged with Micro-XCT (Figures 1 and 2). The bone surface in the mesial root-bone complex is regular and convexly rounded. The channels appearing prominently on the Micro-XCT images are also present and feature red blood cells. Thus, blood vessels in the PDL are continuous with those in bone. The mesial root surface is covered with a regular layer of cementum that broadens towards the apex. On the distal side, the layer of cementum is thin. Furthermore, the root exhibits a number of pits in dentin with a narrow pinch through cementum. Bone in the distal root-bone complex exhibits a strongly pitted surface, with basophilic lines (blue lines) around those pits. Other basophilic-rich regions include cementum resorption sites and cement lines in bone. The cement line is a remnant of the reversal from bone resorption to bone formation during a remodeling process. Hence, cement lines allow us to extrapolate beyond remodeling events. Cement lines can be found everywhere in bone. This shows that although resorption is the dominant process, remodeling is partly executed in the distal complex, as shown in the fluorochrome image (Figure 4 right). The cement line close to the bone surface in the distal root-bone complex, shown in Figure 6((c) lower grey arrow) could be an example for such local remodeling.

Compared to H&E, TRAP staining is specific to mature osteoclasts and more suitable for investigating adaptation of mineralized tissues [24, 46]. It has been shown that the secretion of TRAP by osteoclasts correlates with their resorptive behavior [23, 47], and therefore serves as a selective marker for osteoclastic activity. In our study of the rat periodontium, TRAP-positive cells were almost exclusively observed in the PDL-space of the distal root-bone complex (Figure 7), predominantly close to or in contact with the bone surface. TRAP-positive cells were also found on the surface of the roots, but consistently in lower numbers. These cells were multinucleated like osteoclasts (insert in Figure 7). TRAP-positive multinucleated cells resorbing cementum and dentin were also identified as odontoclasts [48]. 

**Figure 8:**
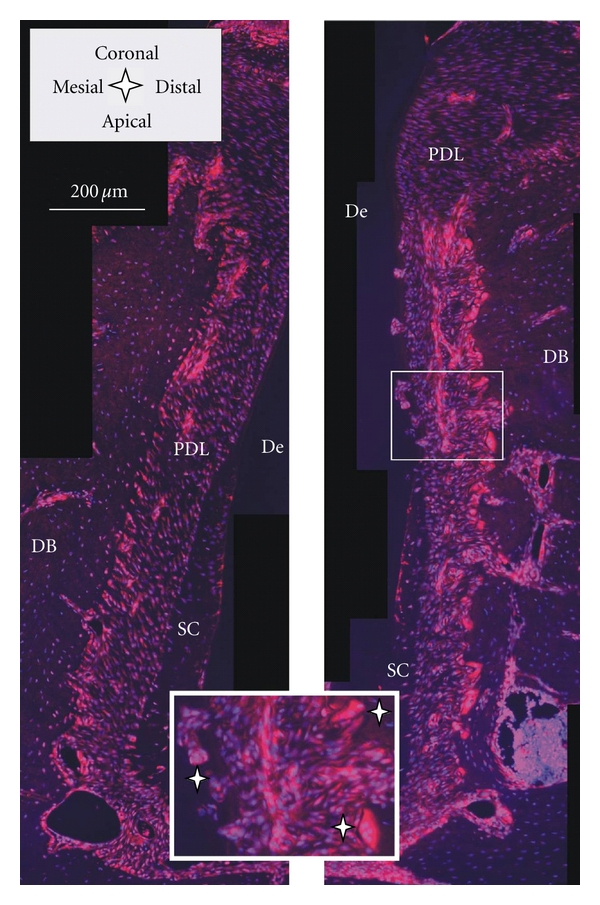
Histological sections immunostained with RANKL; the red RANKL stain is dominant in the PDL close to the bone surface of the distal root-bone complex (right image) compared to the mesial complex (left image); *insert*: note odontoclast on root and osteoclasts on bone (white stars). De = dentin, DB = interdental bone, PDL = periodontal ligament, and SC = secondary cementum.

Specificity, as exhibited by the TRAP stain is also fundamental to immunohistochemistry. It utilizes antibodies to bind to specific antigens of interest. For our study, RANKL and OPN, proteins related to bone remodeling, were chosen. The primary antibody is targeted by a secondary antibody that is bound to a fluorophore. Fluorescence-based immunohistochemistry allowed us to identify the distribution of the desired protein in the tissue. RANKL expression is necessary for differentiation and survival of osteoclasts. An increased number of active osteoclasts is a prerequisite for ongoing resorption due to distal drift. Osteoclastogenesis begins with hematopoietic cells generating mononuclear cells that are stepwise differentiated into mature osteoclasts [41]. Since the step in which mononuclear cells fuse into polykaryons, coincides with TRAP expression, RANKL [49] is recognized to play a significant role. An orthodontic study in rats found that compressed PDL promotes expression of RANKL [25]. In our study, Figure 8 shows that RANKL is upregulated in the compressed PDL of the distal root-bone complex, especially close to the bone-PDL attachment site, as compared to the attachment sites in the mesial complex. The large multinucleated cells in the resorption pits of bone and the root (osteoclasts and odontoclasts) also stained intensely for RANKL (Figure 8 insert). 

**Figure 9:**
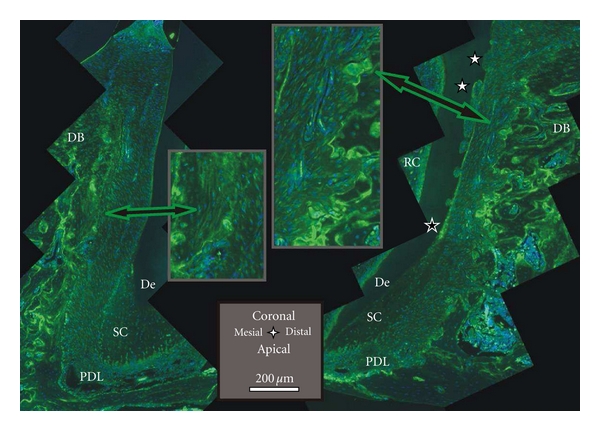
Histological sections immunostained with OPN; the green stain of OPN dominates the distal root-bone complex (right image); note staining on resorption pits and multinucleated cells on bone (right insert), resorption pits on the root (white stars), remodeling in secondary cementum (black star); on the mesial surface of the root (left image) less staining on the bone PDL interface, faint parallel lines in the bulk close to the bone surface in the mesial root-bone complex (left insert), and a single cell (left insert); cement lines stain as bright lines everywhere in the bulk bone. De = dentin, DB = interdental bone, PDL = periodontal ligament, SC = secondary cementum, and RC = root canal.

OPN supports bone remodeling and is produced by osteoblasts, osteoclasts, and a number of other cells. It belongs to the family of small integrin-binding ligand N-linked glycoproteins (SIBLING). The proposed function of OPN in biomineralization [50] is threefold. It promotes cell adhesion of osteoclasts and osteoblasts. It regulates osteoclastic resorption and migration, and was shown to inhibit hydroxyapatite crystal growth by binding to its surface. In our study, most intense staining of OPN was found on the interface of bone and PDL in the distal complex (Figure 9). Its presence is indicated by green lines on the bone surface and green-stained multinucleated cells attached or close to those lines (Figure 9 right insert). The progression from bright to faint staining in the pits on the root surface on dentin, and on cementum, highlights resorption related events. The bulk cementum illustrated an undefined faint stain. Occasionally, brighter staining was found in cementum, specifically at the primary to secondary cementum transition in Figure 9. This could be a sign of recent cementum repair as indicated by Jäger et al. [27]. The progression from bright green to red lines in bone are seemingly related to cement lines, which regularly stain for OPN [28]. The bone surface of the mesial complex neither exhibits bright green lines on the surface, nor osteoclasts complementing lack of TRAP staining in Figure 7. However, close to the bone surface, faint lines were consistently observed (Figure 9 left insert), and could indicate intermittent biomineralization. 

## 4. Conclusions

The functional dentoalveolar complex in rat molars is a highly dynamic system with interactions between functional forces, 3D form, tissues, cells, and biomolecules. The periodontium is an interesting model to study the mechanisms of biomineralization. The inherent distal drift requires ongoing bone formation on the mesial side and resorption on the distal side of the root, to facilitate tooth migration and maintain functional PDL-space. For a better understanding of function-related adaptation, it is necessary to discuss observations at a macroscopic and a microscopic level and correlate them using complementary techniques. With Micro-XCT and post processing of 3D images, we were able to describe the anatomy of the dentoalveolar complex including, approximation of macroscopic occlusion, root geometry, and anisotropy in bone morphology due to the distribution of the microscopic resorption pits on bone and root. With SEM, we increased the resolution and identified structures created by the resorption activity. Attenuation profiles derived from Micro-XCT virtual sections, together with the fluorochrome study, highlighted advancement of the mineralization fronts in the mesial root-bone complex. Fluorochrome labeling pointed out that biomineralization, in relation to repair, can also exist in the distal complex. H&E staining verified structural features from Micro-XCT and SEM studies, and provided a basic understanding of the organic matrix. TRAP allowed for identification of multinucleated cells in the resorption pits of bone and root, found almost exclusively in the distal root-bone complex. Increased RANKL expression as a parallel event to TRAP could be found predominantly close to the distal complex of bone, and to a minor degree at the root surface. We could identify the omnipresence of OPN in the tissue, and related it to its multiple functions in the resorption and remodeling of mineralized tissues. Utilizing a variety of techniques had a synergetic effect to describe and understand the complex dynamic system of the rat periodontium. These results elucidate that load-mediated perturbations and subsequent adaptation of the rat dentoalveolar complex, should acknowledge baseline function based adaptation of bone-PDL-cementum. Local remodeling and modeling related events associated with the physiological distal drift should also be identified before additional experimental variables are imposed.
